# Axial Stretching of Vessels in the Retinal Vascular Plexus With 3D OCT-Angiography

**DOI:** 10.1167/tvst.11.2.21

**Published:** 2022-02-11

**Authors:** Julian Johannes Kuhlmann, Kai Rothaus, Xiaoyi Jiang, Britta Heimes-Bussmann, Henrik Faatz, Marius Book, Daniel Pauleikhoff

**Affiliations:** 1 Faculty of Mathematics and Computer Science, University of Münster, Münster, Germany; 2 Department of Ophthalmology, St. Franziskus-Hospital, Münster, Germany; 3 Department of Ophthalmology, University of Duisburg-Essen, Essen, Germany

**Keywords:** OCTA, cross-section area, vessel

## Abstract

**Purpose:**

The purpose of this study is to describe and quantify the nonpathological axial stretching in the retinal vascular plexus in three-dimensional (3D) optical coherence tomography angiography (OCTA) images.

**Methods:**

The 3D vascular network underneath the inner limiting membrane of OCTA volumes was labeled as ground truth (GT) data. To analyze the cross-section area of the vessels the width and depth of the vessels in the GT data were computed and an elliptical quotient was proposed to quantify the axial stretching.

**Results:**

A total of 21 3D OCTA volumes were labeled. It was found that the vessels in 3D OCTA images are stretched in the direction of the A-Scan by a factor of 2*.*46 ± 1*.*82 with a median of 2*.*24. Furthermore, a larger cross-section area leads to higher axial stretching.

**Conclusions:**

The elliptical shape of the cross-section area of the vessel does not match with the expected pathology of the vascular network in the human eye. Therefore a correction of the volume data before a 3D analysis is recommended.

**Translational Relevance:**

This work gives a systematic insight to the stretched shape of vessels in 3D OCTA images and is relevant for further clinical research analyzing the 3D vascular network.

## Introduction

Since the launch of the first commercial optical coherence tomography (OCT) device in 1996, the importance of the OCT technology increases rapidly in ophthalmology. The OCT technology enables a depth scanning of the retinal layers and provides a three-dimensional (3D) structural representation of the retinal layers. With the introduction of OCT angiography in 2014,^1^ the structural OCT is extended by the representation of “flow.” For each voxel the variation in time of the reflected laser spectrum is measured.^2^ This variation is mainly caused by the travel of erythrocytes in the blood. The resulting OCT angiography (OCTA) volume represents the “flow.”

As one of the most important tasks in biomedical imaging, image segmentation^3^ provides the foundation for quantitative reasoning and diagnostic techniques. Currently, the segmentation of OCTA volume data is realized by a depth-limited two-dimensional (2D) projection within anatomical retinal layers.^4^^,^^5^ A so-called OCTA en face image is generated pixel-wise by aggregating (e.g., averaging or maximum) the flow information of the corresponding voxel stack in the specific retinal layer. The 2D projection procedure has the drawback that it depends on good segmentation of the slabs and loses the information about the 3D structure. Especially in case of retinal diseases the segmentation is not always well defined, even for medical experts. In the case of so-called exudations of the retina, fluid inclusions, or edema, the retina layers are disturbed by elevations of the retina. Therefore we labeled the vascular network of 21 3D OCTA datasets. Numerous medical studies^6^^–^^9^ have shown that the morphology of the vascular network is suitable as biomarkers for various pathological changes of the retina. In this work we first describe the 3D appearance of the retinal vessel plexus of dry age-related macular degeneration cases, which are captured with an OCTA device. Thereby, we focus on the vessels cross section as biomarker to enable the quantification of structural biomarker in further studies. Muraoka et al.^10^^,^^11^ and Rim et al.^12^ characterize the retinal vessels cross-section to be oval in OCT images. As Muraoka et al.^11^ points out, “when the scale ratio was changed to 1:1, vessel sections appeared round.” For this reason, we expect the cross-section area of the average vessel in the human body to be circular. However, we found that the vessels in 3D OCTA images have an elliptical cross-section area even after scale ratio correction, which leads us to the following conclusion: The elliptical cross-section area of vessels in 3D OCTA images is an artifact caused by the imaging technique.

## Methods

### Database

The amount of OCTA image databases with labeled vascular structures is limited; to our best knowledge only Ma et al.^5^ provide an OCTA database with 2D pixel-wise or center-line annotation of the vascular network. In particular there is no database with 3D-labeled vessels, because 3D voxel-wise labels are labor-intensive and difficult to obtain.^13^ Therefore we are going to publish the first 3D OCTA image database with hand-labeled vessels in the retinal vascular complex (Kuhlmann J, et al. Unpublished data), containing a total of 21 volumes provided by the “Augenzentrum am St. Franziskus-Hospital Münster” (available at https://www.augen-franziskus.de/). This study was carried out in accordance with the Helsinki Declaration. There is a positive ethics vote (2016-351-f-S, ethical commission of the “Ärztekammer Westfalen-Lippe,” Münster), and the patients have given written consent to participate. The images were captured with the Avanti Optovue, which has an image size of 304 × 304 × 160 and an axial resolution of 9*.*87 µm in the x- and y-axis and 12.54 µm in the z*-*axis. This is factored in while calculating the results with the elliptical quotient. Note that we refer to the direction of the A-scan as z-axis (depth) and the direction of the B-scan as x- or y-axis (width). First, the vessels were labeled by one of the authors or a student assistant. Then the H.F. refined and corrected the labeling to meet the medical standards. The labeled data will be published open access in the near future. Further details will be published at a later date (Kuhlmann J, et al. Unpublished data).

We segmented the inner limiting membrane (ILM) and the outer nuclear layer (ONL), whereby the ONL was manually refined afterward to guarantee a high-quality region of interest (ROI) for this study. The ROI was set as all voxels in between the detected ILM and ONL. The resulting ROIs contain between ∼1*.*2 million and ∼1*.*6 million voxels, depending on the physiology of the eye. This equals an average ROI thickness of 13 to 17 voxels or ∼0.16 to 0.22 mm. Each dataset has a labeled vascular network of 42.000 to 125.000 voxels (in average 76.000 voxel), which equals 3% to 10% of the ROI. Because the vascular network evolves underneath the ILM, the vessels also evolve in a parallel fashion. The average gradient/slope of the ILM is 5*.*9% ± 3*.*9%, with a median of 5*.*1%. For example, a gradient/slope of 10% equals 5*.*71°, which increases the vessel diameter $d^{'}=\frac{d}{\cos(5.71^{\circ })}$ in the z-axis by 0*.*5%. Thus we can assume that the blood vessels evolve close to orthogonal to the z-axis.

### Determination of Vessel Diameter

To determine the diameters of the vessels, we applied the following 3D method onto the data sets: skeletonize the labeled ground truth data for each skeleton voxel, and save all vessel diameters in a list:

(a) determine the spherical neighborhood with a diameter parameter *d_sphere_* around the skeleton voxel;
(b) count the number of voxels n inside the sphere that are labeled as vessel and are connected to the skeleton voxel;
(c) divide the number of voxels by the diameter of the sphere to get the cross-section area of the vessel $A_{c}=\;\frac{n}{d_{sphere}}$ .

**Figure 1.**
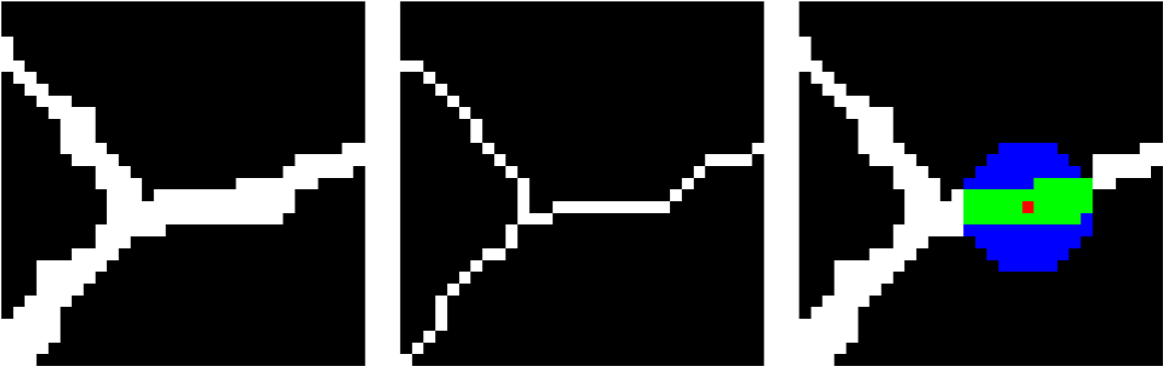
2D example to determine the vessel width. *Left*: Labeled ground truth. *Middle*: Skeletonized ground truth. *Right*: Illustration for determining the vessel diameter in 2D with the current skeleton pixel (*red*), the circular area (*blue* and *green*), and the overlap of circle and vessel (*green*). Thirty-seven overlapping pixels with a circle diameter of *d_circle_* = 11*px* resulting in a vessel diameter of $d_{v}=\frac{n}{d_{circle}}=3.36px$.

Note that this algorithm also works similarly in 2D, when using a circle instead of a sphere and the width of the vessel instead of the cross-section area (Fig. 1).

**Figure 2.**
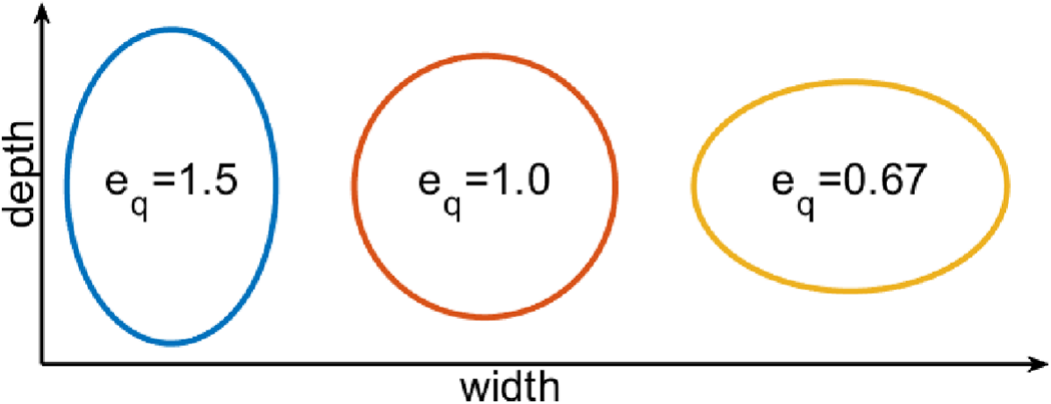
Exemplary illustration of the proposed elliptical quotient.

Under the assumption that the cross-section area for 3D vessels is circular, we can calculate the vessel diameter as:

(1) $$d_{v}=2\cdot \sqrt{\frac{A_{C}}{\pi }}.$$

We chose the circle/sphere as the neighborhood because it is rotation invariant. Thus our method to determine the vessel diameter is independent of the vessel orientation.

### Elliptical Quotient

Here we will propose an elliptical quotient to describe the axial stretching or contraction of a tubular structure (Fig. 2). The general definition of an ellipse, with $\bar{a},\bar{b}>0$ is given by:

(2) $$\frac{x^{2}}{{\bar{a}}^{2}}+\frac{y^{2}}{{\bar{b}}^{2}}=1,$$

where *a* is the semi-major axis and b is the semi-minor axis, thus $\bar{b}\leq \bar{a}$ holds. Then the eccentricity of an ellipse is defined as:

(3) $$ɛ=\sqrt{1-{\left(\frac{\bar{b}}{\bar{a}}\right)}^{2}}.$$

With $\bar{a}=\bar{b}$ we have a circle and therefore the eccentricity in 0. The eccentricity describes the axial stretching of the ellipse in the semi-major axis $\bar{a}$. The label of the semi-major and semi-minor axis swaps every time when a vessel switches from being stretched in one axis to being stretched in the other.

Thus the use of the eccentricity to measure the contraction and stretching of the vessels is impractical. Therefore we define the elliptical quotient as:

(4) $${ɛ}_{q}=\frac{b}{a}=\sqrt{1-{ɛ}^{2}},$$

where a is the diameter in one axis of the coordinate system and b is in the other. The elliptical quotient is the length ratio of the axis of the vessel, which is larger than 1, if *b* > *a* and < 1 otherwise.

### Computation of the Elliptical Quotient on OCTA Vessels

The x- and y-axis in 3D OCTA images captured with the Avanti are similar in term of resolution and behavior. The z-axis stands out because of the imaging process, because it depends on the penetration depth of the OCTA. Because the vascular network evolves underneath the ILM, the vessels also evolve in a parallel fashion. Therefore we set the width of the vessels in the x/y plane as 2·*a* and the depth of the vessel in the z-axis as 2 *b* because the vessels are approximately orthogonal to the z axis.

We determine the depth *d_depth_* in the z-axis by counting the amount of ground truth voxels above and below the skeleton voxels, similar to the process when creating en face images. The influence of the slope of the vessels can be ignored since 95% of the ILM has a gradient *<* 12*.*9% (equals 7*.*35°), which would increase the depth by the factor of $\frac{1}{\cos(7,35^{\circ })}=0.83\%$. The width *d_width_* can be derived from the cross-section area:

(5) $$d_{width}=\frac{4\;\cdot A_{c}}{\pi \cdot d_{depth}},$$

by using the fact that the area of an ellipse equals $\pi \cdot a\cdot b=\frac{1}{4}d_{depth}\cdot d_{width}\cdot \pi$. A less complex approach would be to use a bounding box to determine the width and depth. But we preferred to use the cross-section area approach because of the lower sensitivity to wrongly labeled voxels.

## Results

The data were labeled and analyzed using MATLAB (version 2019a). To compare and visualize the data we performed descriptive statistics, including frequency analysis, Cohen's kappa, calculation of the mean and boxplots using the MATLAB built-in routine (available at https://de.mathworks.com/help/stats/boxplot.html). We split the results into two subsections: The first presents the voxel-based results, the second uses the elliptical quotient to quantify the axial stretching.

### Analysis of Vessel Width and Depth

**Figure 3.**
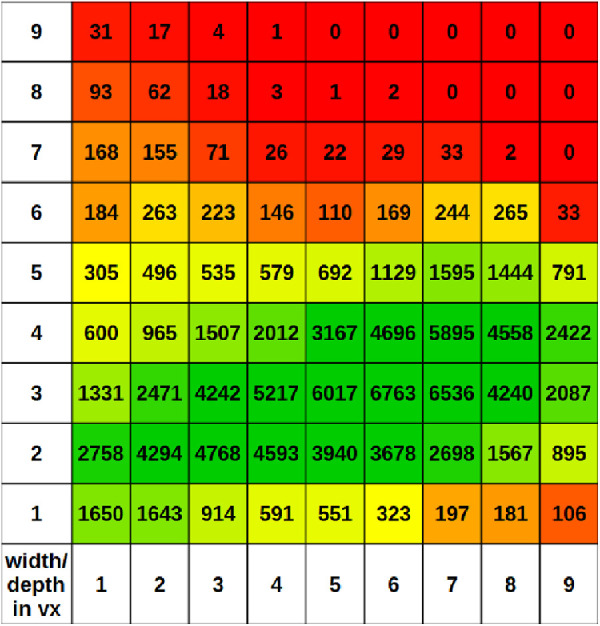
Distribution of width (rows) and depth (columns) in voxels for all skeleton voxels. *d_depth_* = 1*vx*, since we cannot label or measure a depth smaller than one voxel. Since the OCTA image is not isotropic, this effect is further increased by the resolution of 9.87 µm per voxel in the x- and y-axis and 12*.*54 µm per voxel in the z-axis. Therefore, we will use the elliptical quotient as introduced in section 2.3 to better describe this behavior.

After skeletonizing the vascular network of the 21 data sets a total of 117.035 skeleton voxels remained to analyze the axial stretching in the data. Each data set has between 3364 and 7756 skeleton voxels. We choose the sphere diameter parameter *d_sphere_* to be 9*vx* (voxel). The resulting average width is 3*.*08*vx* ± 1*.*08*vx*, the average depth is 5*.*49*vx* ± 2*.*61*vx*, and the average cross-section area for the vessel is 13*.*86*vx* ± 8*.*73*vx*. In Figure 3 we can see the overall distribution of the depth and the width of the vessels rounded to the next full voxel. We found a shift toward vessels with a depth higher than the width. Two thirds of all skeleton voxels belong to a vessel with *d_width_* ≤ 4*vx* and a *d_depth_* ≥ 4*vx*. Overall, in 76% of the skeleton voxels the vessel width in *vx* is smaller than the depth in *vx*, in 12% they are equal, and in 12% the width is greater than the depth in *vx*. The latter fact is mostly due to the vessel width.

### Elliptical Quotient

Using the resolution of the axes, we found that an average width is 30.4 µm ± 10.7 µm, an average depth is 68.8 µm ± 32.7 µm, and an average cross-section area for the vessel is 1715 µm^2^ ± 1080 µm^2^. For a vascular network we expect an elliptical quotient close to 1. Here, the average elliptical quotient for the retinal vascular network is 2*.*46 ± 1*.*82 with a median of 2*.*24. This implies that on average retinal vessels in OCTA images are more than two times wider in the z-axis than in the x and y plane.

**Figure 4.**
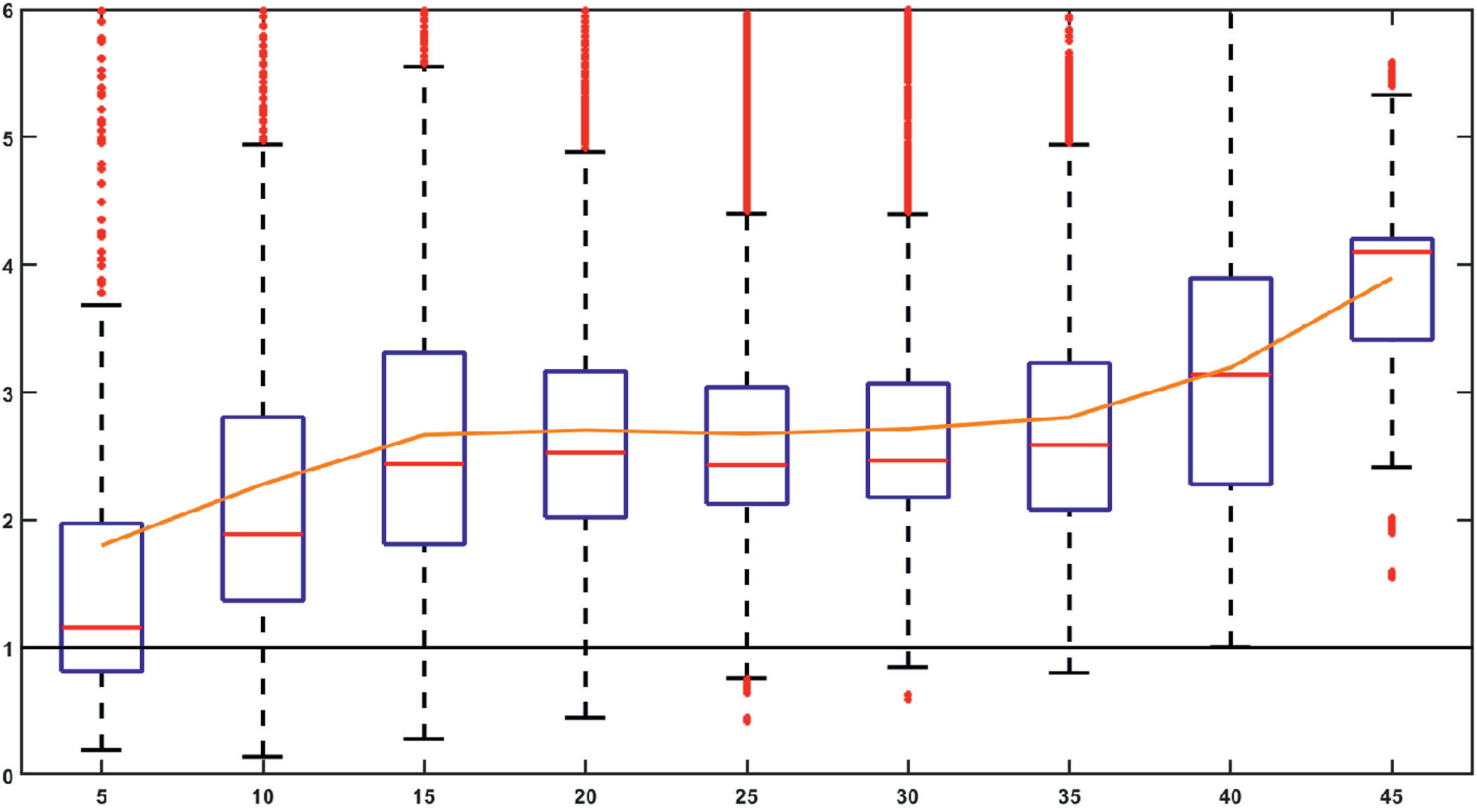
*Box plot* and the mean value (*orange curve*) of the elliptical quotient ε_*q*_ with the cross-section area of the vessel in voxels (rounded up) in the x-axis, the elliptical quotient in the y-axis. *Black baseline* for expected ε_*q*_ = 1.

As illustrated in Figure 4 we found that higher vessel cross-section area leads to a higher elliptical quotient. The elliptical quotient is lowest for vessels with a small cross section area. This can be explained by the fact that for a cross-section area with ≤5 voxels, the vessels have a shape with a width or depth smaller <3 voxels, which limits the range of the elliptical quotient.

**Figure 5.**
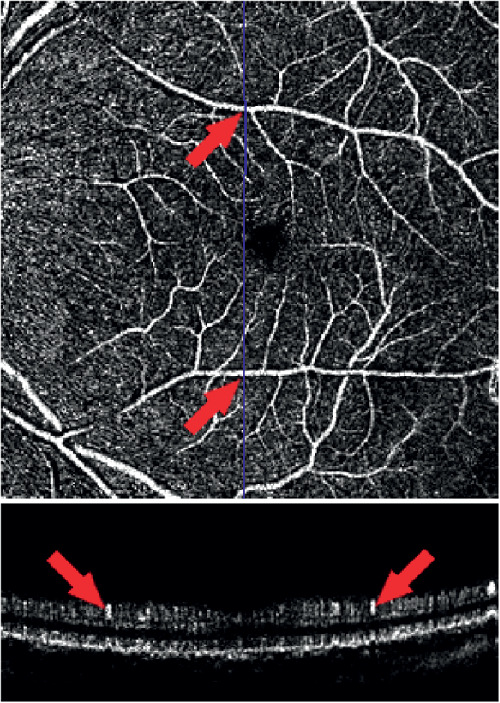
*Top*: z-slice (parallel to the ILM) of the vascular network. *Bottom*: B-scan of the OCTA (corresponds to the *blue line* in the top image) with two examples for vessels with a high elliptical quotient (left ε_*q*_ = 4.14, right ε_*q*_ = 4.19).

In Figure 5 we can see two examples for vascular network with a high elliptical quotient ε_*q*_ > 3. As illustrated, these two vessels run throughout the whole image with an elliptical quotient ranging between 3.2 and 4.4. Thus the visual observation confirms the results from the analysis above.

For further analysis we used Zana's algorithm to rule out the subjective influence on the effect. Therefore we calculated a pairwise Cohen's κ using the implementation from G. Cardillo (available at https://github.com/dnafinder/Cohen) to show that the Zana algorithm is a viable algorithm to segment the vascular network (Table 1).

**Table 1.** Pairwise Cohen's κ for the 21 Images From Our Optovue Database for Different Observers

Medical Expert	Nonexpert	Zana	Observed Agreement	Random Agreement	Cohen's κ
X	X		0.9998	0.9892	0.9835
X		X	0.9969	0.9919	0.6227
	X	X	0.9968	0.9917	0.6118

**Figure 6.**
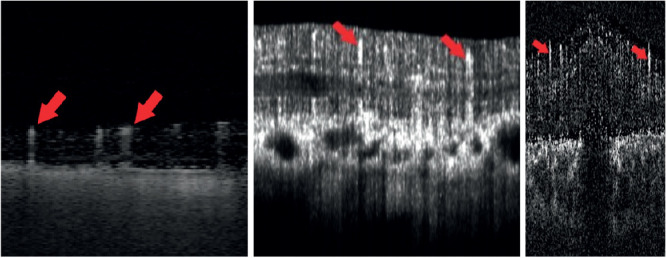
Comparison of three other OCTA device manufacturers: Slice of a 3D OCTA images captured with the “Zeiss PlexElite” (*left*, 10 µm in the x-axis and 19*.*5 µm in z-axis per voxel), the “Heidelberg Engineering's SPECTRALIS OCTA” (*middle*, 19*.*5 µm in the x-axis and 12*.*2 µm in z-axis per voxel), and the “ZEISS CIRRUS OCT with AngioPlex” (*right*, 11 µm in the x-axis and 3.9 µm in z-axis per voxel). Note the elongated, elliptical shaped, vascular structures marked by the *red arrows* that are higher than the expected axial stretching caused by the differences in axial resolution.

Furthermore, we did an interobserver analysis by calculating the mean, the standard deviation, and the median. The observers are the expert labeling, the nonexpert labeling, and the results of applying the Zana algorithm^14^ to get a segmentation of the vessels. Furthermore, we have a subset of two images, for which we also have an image captured with the Zeiss PlexElite at the same day for the same patient (Table 2). Now we can make a transitive argumentation chain: Because the Zana segmentation is similar to the human segmentation for the Optovue images, the Zana segmentation for the PlexElite is also an adequate segmentation for the vascular network. From these results and the values from Tables 1 and 2, we can draw two conclusions: First, the distribution characteristics are similar for different manufacturers, and, second, the analyzed effect is not observer and noise independent. Additionally, we visualized the captured images for two more machines (Fig. 6). We can see in these images that they indicate a similar behavior.

**Table 2.** Elliptical Quotient for Different Observers

Observer	Mean	STD	Median
Optovue Medical Expert	2.46	1.82	2.24
Optovue Nonexpert	2.42	1.56	2.22
Optovue Zana	2.50	2.85	2.10
Optovue Medical Expert (subset)	2.85	1.89	2.63
Optovue Nonexpert (subset)	2.90	1.80	2.69
Optovue Zana (subset)	2.40	2.90	1.95
PlexElite Zana (subset)	2.64	3.19	2.13

## Discussion

To the best of our knowledge, there is no literature that analyzes the shape of the cross-section area of the vascular network in 3D OCTA images. There are some limitations to the algorithm which determines the vessel diameter: The vessel diameter for last skeleton voxels at the end of the vessel is underestimated by up to 50%. But this affects only a small share of the skeleton voxels. Also, there is a slight overestimation at branching points. But these two conditions only hold true for a low percentage of the overall voxels. Furthermore, heavily tortuous vessels in the x-y plane within the circle/sphere area leads to an overestimation of the vessel diameter. But choosing a sphere diameter smaller than the curve radius of the vessel will prevent this. All these of limitations might lead to a slightly higher cross-section area, which implies a lower elliptical quotient, and therefore our findings are not artificially increased by the limitations above.

We will discuss three different hypotheses that could cause this effect:

1. This is the true histology of the vascular network.
2. The capturing technique artificially increases the vessel diameter in the z-axis.
3. The algorithm to compute the OCTA image causes the increased vessel diameter in the z-axis.

Because our findings do not match the observation from histologic examination of the vessels in the retinal vascular plexus, we can eliminate option 1. Therefore there must be a different root to this elliptical deflection of the vessels in OCTA images.

**Figure 7.**
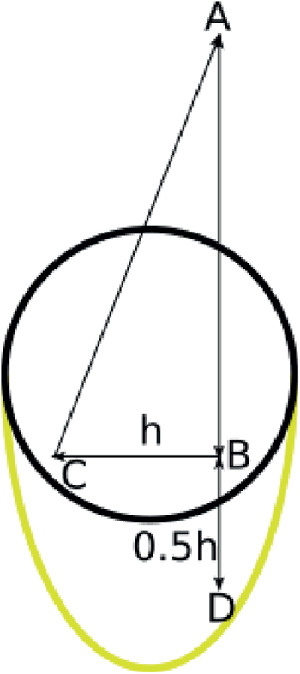
Simplified, not true to scale, representation of the OCT/OCTA imaging technique for a vessel (*black circle*) with a double reflection in lateral direction.

In th following we present a possible explanation during the capturing process of the image to explain this unexpected behavior. Our idea implies that the laser is reflected more than one time when hitting a blood vessel. Usually, we assume that the light from the OCTA device in position A (Fig. 7) penetrates the tissue and is then reflected by a red blood cell inside a vessel in position B, back toward position A. But if the laser is reflected two times, one time in position B and one time in position C, the total path length is increased by the length *h* (distance between B and C). This is then recognized as a virtual penetration depth, which is by *h*/2 greater than the real penetration depth (distance between A and B). This leads to axial stretching in the z-axis (the depth) and therefore an elliptical cross-section area for the vessels. Furthermore, thicker vessels lead to a higher chance of a double reflection and therefore is consistent with the observation that a higher vessel cross-section area leads to a higher elliptical quotient. A reflection in lateral axis explains an increased elliptical quotient up to ε_*q*_ = 1.5 because the increase of the penetration depth in limited by 50% of the vessel diameter. Because we observe an elliptical quotient of ε_*q*_ = 2.46, we have to assume that this is caused by longitudinal reflections or a combination of both. This is a possible explanation for option 2. So far, we are not able to validate this approach. Further validation with a retina phantom might be an interesting idea. But this would go beyond the scope of this work. Because we do not have access to the code of the manufacturer, we cannot examine the third option.

For the analysis of 2D en face images, this effect can be neglected because the influence of a deflection in the z-axis does not influence the maximum calculations in the z-axis. However, if one is interested in the cross-section area of the flow, the overall flow volume, or other 3D-dependent measures like a 3D fractal dimension analysis, we recommend looking for methods to correct the deflection of the vessels.

## Conclusion

In this article we analyzed the axial stretching of vessels in the retinal vascular plexus in 3D OCTA images. Since the shape of the cross-section area does not match with the expected pathology and other manufacturer show similar behavior, we provide an important contribution towards the development of 3D biomarkers for clinical use. Future studies should aim to verify our explanation approaches, further analyze the behavior on other OCTA-machines, and implement 3D biomarkers that use the findings presented in this article.
